# Perception and Acceptance of Using Different Generic Types of COVID-19 Vaccine, the “Mix-and-Match” Strategy, in Saudi Arabia: Cross-Sectional Web-Based Survey

**DOI:** 10.3390/ijerph192113889

**Published:** 2022-10-26

**Authors:** Afnan Alqurashi, Enas A. Sindy, Heba Dosh, Sumaya Z. Khayat, Lujain M. Alqarna, Wafa M. Sodagar, Mohammed Samannodi, Hassan Alwafi, Emad Salawati, Mohammed A. Almatrafi, Rakan Ekram, Rehab M. Bagadood, Radi Alsafi, Hamza M. Assaggaf

**Affiliations:** 1 Independent Researcher, Makkah 4192, Saudi Arabia; 2 Collage of Medicine, Umm Al-Qura University, Makkah 24382, Saudi Arabia; 3 Department of Family Medicine, Faculty of Medicine, King Abdulaziz University, Jeddah 22254, Saudi Arabia; 4 Department of Pediatrics, Umm Al-Qura University, Makkah 24382, Saudi Arabia; 5 School of Public Health and Health Informatics, Umm Al-Qura University, Makkah 24382, Saudi Arabia; 6 Department of Laboratory Medicine, Faculty of Applied Medical Sciences, Umm Al-Qura University, Makkah 24382, Saudi Arabia

**Keywords:** SARS-CoV-2, COVID-19 vaccine, mix-and-match

## Abstract

**Background:** Soon after the COVID-19 pandemic was declared, a pharmaceutical company expressed rapid interest in developing a safe and effective vaccine candidate to contain the spread of SARS-CoV-2 infections. The FDA approved the Pfizer-BioNTech, AstraZeneca, Moderna, and Janssen vaccines. Here, we investigated the attitude and acceptance of using different generic types of COVID-19 vaccines in Saudi Arabia. **Methods:** This study is a cross-sectional study using an online survey conducted in Saudi Arabia from the 19th of October to the 6th of December 2021. The questionnaire was distributed using social media platforms such as Twitter, WhatsApp, and Facebook. The inclusion criteria to participate in this study were adults who live in Saudi Arabia (Saudis or non-Saudis) and had two doses of COVID-19 vaccinations. **Result:** 3486 participants were included in this study, and 67.5% of the participants had side effects after the first dose. Similarly, 66.7% of the study participants had side effects after administering the second dose. Our data showed that most participants were unsure if the heterologous COVID-19 vaccination could cause severe side effects. In addition, 47.6% of the participants refused to receive a different generic type of COVID-19 vaccine due to fear of health problems. However, most participants obtained information regarding COVID-19 vaccination from the Saudi Ministry of Health. **Conclusions:** We found a low level of acceptance for receiving different generic types of vaccines if the participants had a choice. Therefore, plans should focus on increasing the acceptance level among the Saudi population through official platforms such as the Saudi Ministry of Health and private clinics.

## 1. Introduction

COVID-19, caused by (SARS-CoV-2), is a beta-coronavirus strain that causes atypical respiratory disease [1], commonly associated with high-grade fever; chills; and respiratory symptoms such as cough and dyspnea; sore throat; and other symptoms such as persistent tremor, muscle pain, headache, ageusia, and anosmia [2].

The evolution of the SARS-CoV-2 strain of the human coronavirus has brought a new pandemic worldwide. SARS-CoV-2 was declared a public health emergency of international concern on 30 January 2020. As of 27 March 2022, over 479 million confirmed cases and over 6 million deaths had been reported globally [3]. According to a recent study, COVID-19 infection was found in most Saudi Arabian provinces in March 2020, with a peak in June–July 2020 [4]. Governments have applied preventive measures to stop the spread of COVID-19 by implementing public health measures such as hand hygiene, personal protective equipment and safe waste management, environmental cleaning, social distancing, and widespread vaccination programs [5].

Vaccination protects against SARC-CoV-2 infection and prevents severe disease [6]. Evidence suggests that, after 12 days of the first vaccination dose, there is a reduction in infection [7]. Moreover, fully or partially vaccinated individuals were less likely to experience a significant complication and fewer hospital stays [6]. The United States Food and Drug Administration (FDA)-approved vaccines include Pfizer-BioNTech, AstraZeneca, Moderna, and Janssen COVID-19 immunization [8]. On 12 October 2021, the FDA approved the utilization of a heterologous “blend or match”. Accordingly, several models of vaccine utilization were proposed [9].

Over the past decade, various studies have demonstrated that a heterologous prime-boost regimen can be more immunogenic than a homologous prime-boost regimen [10,11]. The Moderna, Janssen, and Pfizer-BioNTech vaccines are approved for a booster dose for those classified as a high-risk group (65 years and older) [12,13]. Mixing COVID-19 vaccinations targets raises the protective efficacy (a better immune response than a single vaccine regimen) and allows people to become used to the available vaccines [9]. Based on the FDA’s approval of the mix-and-match strategy of the COVID-19 vaccination program, limited research has been conducted to measure the acceptance of the mix-and-match of COVID-19 vaccination [14]. Our study aimed to investigate the Saudi public attitude and acceptance of taking two different types of COVID-19 vaccines between the first and second doses.

## 2. Methods

### 2.1. Study Design

A quantitative cross-sectional study was conducted in Saudi Arabia from the 19th of October to the 6th of December 2021 using a web-based self-administrated questionnaire to explore public attitudes and acceptance of taking two types of COVID-19 vaccines between the first and second doses. The inclusion measures to participate in this study were all adults (males and females) aged 18 years or above and those living in Saudi Arabia (Saudis or non-Saudis) who obtained two doses of vaccines.

### 2.2. Sampling Strategy

A random convenience sampling strategy was utilized to recruit possible eligible participants in the study. For example, an invitation containing a survey link was posted on social media platforms. This invitation was reposted on the platforms to make it visible to a larger population and to increase the response rate.

As all participants agreed to participate in the study, they were not required to provide written informed consent. However, the study aims, objectives, and inclusion criteria were clearly explained at the beginning of the survey invitation letter. Finally, participants accessed the questionnaire and completed it if they agreed to participate.

### 2.3. Survey Instrument

Since this study’s aim and objectives were considered new when the study was conducted, a validated questionnaire had not existed. Therefore, three experts (a virologist, an immunologist, and a health psychology consultant) at Umm Al-Qura University developed a questionnaire to investigate the public acceptance and attitudes toward receiving two different generic types of COVID-19 vaccine.

A pilot study using the developed questionnaire was conducted on 30 participants in Saudi Arabia, including Saudis and non-Saudis, who met the inclusion criteria for the study. The inclusion criteria to participate in this study were ≥18 years, living in Saudi Arabia (Saudis or non-Saudis), and having two doses of COVID-19 vaccinations. Participants were asked to provide feedback on the clarity and comprehension of the questionnaire and if any questions were challenging to understand. Participants in the pilot study confirmed that the questionnaire was considered easy to understand and comprehensive. Questions that were found difficult to understand have been adjusted according to the participant’s suggestions. Finally, the questionnaire was evaluated by three experts (an infectious disease consultant, a family medicine consultant, and an epidemiologist). They found that the questionnaire was clear, concise, and comprehensive for answering the study’s aim and objective.

The final version of the questionnaire consisted of 29 items. These 29 questionnaire items comprised three sub-scales questions: The first section contained nine questions about demographics. The second section contained eight questions about the first and second dose vaccine types and their side effects. The third section contained questions about participants’ perceptions and attitudes toward a mix-and-match vaccine strategy (division A = 10, division B = 2).

## 3. Result

### 3.1. Participant’s Characteristics

Among 4044 participants, 3486 were included in our study; however, 558 participants were excluded for not meeting inclusion criteria (≥18 years, living in Saudi Arabia (Saudis or non-Saudis), and having two doses of COVID-19 vaccinations) (Table 1). All of them had two doses of COVID-19 vaccinations. The study’s sample (*n* = 3486) had received two doses of COVID-19 vaccinations. Two-thirds of the participants were female, 2436 (69.9%), and 1050 (30.1%) were male. Most of the respondents, 3242 (93%), were Saudi. More than half of the participants (57.7%) were between 18 and 29 years old, whilst 670 (19.2%) were 30–39 years old. Moreover, 1805 (51.8%) of the sample were single, and 1554 (44.6%) were married. Furthermore, 1098 (31.5%) participants were from the western region, while 976 (28%) were from the eastern part. A total of 1998 (57.3) had a bachelor’s degree, and 833 (23.9%) had attended high school. Additionally, 1382 (39.6%) were students, whereas 1100 (32.6%) were employees.

### 3.2. First and Second Doses of COVID-19 Vaccination and Their Side Effects

Most individuals (76.3%) received the same brand of vaccination for their first and second doses. The generic types for the first dose among the study participants included Pfizer (74.4%), AstraZeneca (24.9%), Moderna (0.5%), and Janssen (0.2%), and the second dose included Pfizer (78.3%), AstraZeneca (18.2%), Moderna (3.2%), and Janssen (3.2%).

In total, 67.5% of the cases had side effects after the first dose (Table 2). Similarly, 66.7% of the study participants had side effects after administering the second dose (Table 3). There were common side effects after both first and second doses, such as pain at the injection site (56.2% and 50.8%, respectively) in about half of the subjects and fever in about thirty percent. Furthermore, 29.8% of respondents experienced fatigue after the first dose, and 28.1 had muscle pain following the second dose. Despite that, nearly thirty per cent of the subjects did not have any symptoms following the first or second doses.

Regarding their susceptibility to COVID-19 infection after immunization, 3386 (97.1%) of individuals claim that they did not become infected after receiving the second vaccination. However, 40 participants (1.1%) did so less than two weeks after receiving the second dose, and 60 participants (1.7%) became infected after two weeks from receiving the vaccine.

### 3.3. Perception and Attitude toward the “Mix-and-Match” Strategy of COVID-19 Vaccines

When observing the participant’s responses, we found that 43.7% of them were neutral toward the idea that mixing vaccine types causes severe side effects. At the same time, 42.5% of participants were equally concerned that receiving different generic types of COVID-19 vaccines is ineffective. Interestingly, if many people take different generic types of COVID-19 vaccine, 29.1% of the participants will follow their lead. In contrast, 41.7% will take it if their family doctor advises them, and 47.7% will likely take it if there is no alternative. In addition, 44.4% accepted taking two different types of vaccines if it was mandatory. Almost 45% of the participants will receive the vaccine only if provided with enough information on its safety.

**Figure 1:**
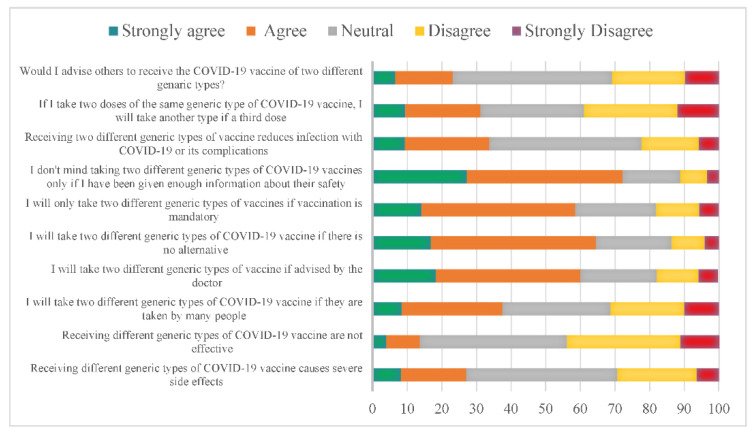
Perception of using various generic types of COVID-19 vaccines, the “mix-and-match” strategy, in Saudi Arabia. The X axis represents the percentage of the participants.

Furthermore, 44% were unsure if mixing vaccines would reduce infection rates or its complications. However, 26.9% disagree with choosing another type, for example, if a third dose is available after two doses of the same kind, and 30% were neutral while 46.1 were uncertain if they would advise others to receive a different type of vaccine (Figure 1). However, our data showed no strong correlation between the acceptance of mixing and matching COVID-19 vaccines with participants who received COVID-19 immunization (Supplementary Table S1).

**Figure 2:**
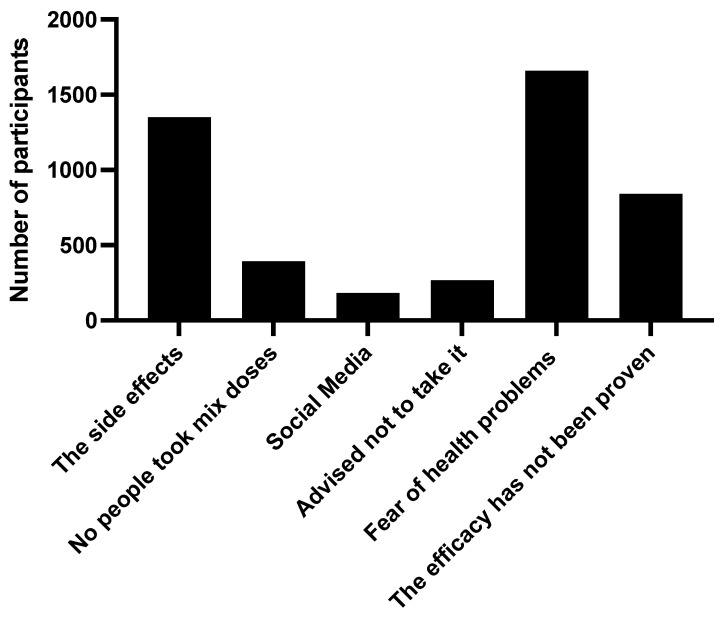
The reasons for participants to use different generic types of COVID-19 vaccines, the “mix-and-match” strategy, in Saudi Arabia.

Upon investigating the reason for the refusal of mixed types of vaccines, a fear of health problems arising from taking it (47.6%) was found. There were around 38.7 subjects who refused to take it considering the side effects, and 24.1 would not take it as long as efficacy has not been proven by international research (Figure 2).

**Figure 3:**
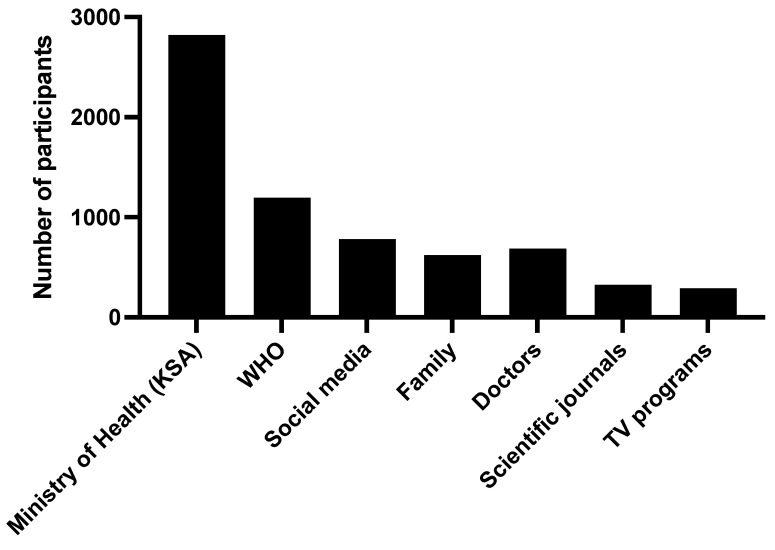
The sources of the information regarding COVID-19 vaccinations.

Regarding the source of information on vaccination among the participants, the vast majority (80.9%) referred to the ministry of Heath as a source of information, in addition to the World Health Organization (34.3%); social media (22.4%); and other sources such as consulting a doctor, family and close friends, scientific journals, and TV programs (Figure 3).

## 4. Discussion

Vaccinations are considered one of the most effective methods of preventing infectious diseases [15]. We aimed to investigate the public perception and acceptance of taking two different generic types of COVID-19 vaccine between the first and second doses. Our sociodemographic data demonstrated that a highest number of participants were young adults between 18 and 29 years old since most of the Saudi population is within this age group [14]. Most of them have bachelor’s degrees (57.3%) since they are supposed to be well-educated, and many adhered to the prevention measures to avoid COVID-19 infection.

Different types of COVID-19 vaccines have been used among participants who received any initial two-dose combinations approved by the government. Moreover, about 76.3% of participants received both doses from the same type of COVID-19 vaccine, while 22.5% received both doses from different generic types of COVID-19 vaccine. Convergent findings in a study recently published in Canada found that around 79% of participants received both doses from the same brand and type of vaccines, mostly the Pfizer and Moderna vaccines. In comparison, 16% of them received two doses of the vaccine from different brands [14].

The participants who received heterologous vaccines considered the vaccine’s side effects and long-term effects. However, a recent study published in the United States assessing the safety of heterologous booster vaccinations for all COVID-19 vaccines found that the rate of the side effects was similar, regardless of the type of booster received. Still, the side effects reported were higher following a Moderna COVID-19 vaccine booster [16]. Our study showed that side effects were similar between the first and second doses. However, another study revealed that the side effects were higher after the second dose than the first dose [14].

Our study found that the main reason to reject mixing doses of vaccines is fear of health problems or severe side effects. Moreover, the population’s acceptance of the “mix-and-match” strategy in our study is obligation or lack of choice. However, some studies extensively describe the health problems and side effects of different types of COVID-19 vaccination [7,17]. For example, a study reported that the side effect profile in Pfizer is like that after homologous vaccination with AstraZeneca [18]. Therefore, public awareness should be raised about the safety and efficacy of mixing the doses of the COVID-19 vaccine.

One reason for the lacking acceptance and awareness of mixing between COVID-19 vaccinations depends on the sources of information about COVID-19 vaccines [19]. Regarding the origin of vaccine information, our study showed that most participants obtained vaccine information from the Ministry of Health and its official platforms.

Therefore, the variety of sources declaring the importance of COVID-19 vaccination and the safety of heterologous COVID-19 vaccines, such as media, social media, and brochures in the local clinics, should be increased.

### Strength and Limitations

This study has several strengths. First, to the best of our knowledge, this study is the first study in the Middle Eastern region to explore public attitudes towards the mix-and-match vaccine strategy against COVID-19. Second, the research employed a questionnaire developed to suit the people living in Saudi Arabia and the purpose of the research aims and objectives, which ensured the study’s quality and the findings reported. However, there are some limitations. It is a cross-sectional observational study conducted through a web-based online survey (Twitter, Snapchat, and WhatsApp).

The study design itself could acquire several limitations. First, the study is subject to recall or implicit bias because participants may be influenced by the news or social media stories about the pandemic. Moreover, conducting a web-based survey using social media could affect the study’s outcome compared to other collection methods such as advertising, for instance, daily newspaper, mail, etc. [20,21,22,23]. Nevertheless, the study was conducted almost immediately after the Saudi government announced that having two doses of the vaccine is obligatory, which might have reduced any potential recall bias. Furthermore, it is assumed there are no benefits for the participants to lie about vaccination history and their attitudes towards the mix-and-match vaccine strategy; the larger sample size could have mitigated the outliers’ response effects. Second, employing a convenience sampling strategy by posting an online survey on different social media platforms might downsize the generalizability of the study. However, reposting the invention link several times on these social media platforms and the study’s large sample size neutralized the effect of using a convenience sampling strategy. Additionally, using social media platforms and a web-based self-administered questionnaire was a suitable strategy during the pandemic.

## 5. Conclusions

The findings of this research have significant implications for the scientific community and public health officials because of the possible continuation of the mix-and-match strategy for COVID-19 vaccination. We found a low level of acceptance for receiving different generic types of vaccines (mix-and-match strategy). This acceptability level increases if it is accompanied by an increase in (mix-and-match) vaccine recipients, doctor advice, and the absence of alternatives. Therefore, to improve the acceptance level among the Saudi population for the mix-and-match strategy, awareness programs by the Ministry of Health, health care workers, and champions and leaders should be carried out to increase the acceptance level of the mix-and-match strategy. Further research should also be conducted to evaluate the efficacy and safety of the mix-and-match COVID-19 vaccine strategy.

## Figures and Tables

**Table 1:** Baseline characteristics of participants (*n* = 3486).

Variables		*n*	%
**Gender**	female	2436	69.9
	Male	1050	30.1
**Nationality**	Saudi	3242	93.0
	Non-Saudi	244	7.0
**Age**	18–29	2013	57.7
	30–39	670	19.2
	40–49	513	14.7
	50–59	247	7.1
	60<	43	1.2
**Social Status**	Widower/widow	33	0.9
	Single	1805	51.8
	Married	1554	44.6
	Divorcee	94	2.7
**Region**	South	398	11.4
	Eastern	976	28.0
	North	617	17.7
	Western	1098	31.5
	Central	397	11.4
**Educational Level**	Primary school	33	9.0
	Secondary school	87	2.5
	High school	833	23.9
	Bachelors	1998	57.3
	Diploma	343	9.8
	Postgraduate	192	5.5
**Job**	Student	1382	39.6
	Free work	152	4.4
	Unemployed	852	24.4
	Employed	1100	32.6
**Do you take both doses of vaccine from the same type?**	No	783	22.5
	I don’t know	44	1.3
	Yes	2659	76.3
**Type of vaccine in the first dose**	AstraZeneca	867	24.9
	Janssen	7	0.2
	Moderna	18	0.5
	Pfizer	2594	74.4
**Side effects from the first dose**	No	1132	32.5
	Yes	2354	67.5
**Type of vaccine in the second dos**	AstraZeneca	636	18.2
	Janssen	9	0.3
	Moderna	110	3.2
	Pfizer	2731	78.3
**Side effects from the second dose**	No	1161	33.3
	Yes	2325	66.7
**Did you get a SARS-CoV-2 infection after the second dose?**	No	3386	97.1
	Yes, <two weeks	40	1.1
	Yes, >two weeks	60	1.7

**Table 2:** Side effects after the first dose (*n* = 2354).

	N	AstraZeneca	Janssen	Moderna	Pfizer	%
**Fever**	1213	547 (45.09)	2 (0.16)	12 (0.98)	652 (53.77)	34.8
**Pain at the injection site**	1958	502 (25.63)	2 (0.1)	11 (56.17)	1397 (71.34)	56.2
**Heat and swelling at the injection site**	607	204 (33.61)	2 (0.32)	6 (0.98)	383 (63.09)	17.4
**Fatigue**	1040	408 (39.23)	2 (0.19)	4 (0.38)	611 (58.75)	29.8
**muscle pain**	944	376 (39.83)	1 (0.1)	5 (0.52)	550 (58.26)	27.1
**Headache**	871	350 (40.18)	0	7 (0.8)	492 (56.48)	25.0
**Diarrhea**	85	25 (29.41)	0	3 (3.52)	46 (54.11)	2.4
**Rash**	34	10 (29.41)	0	1 (2.94)	23 (67.64)	1.0
**Stroke (heart, lung, brain)**	4	0	0	0	4 (100)	0.1
**No symptoms**	1009	153 (15.16)	3 (0.29)	4 (0.39)	847 (83.94)	28.9

**Table 3:** Side effects after the second dose (*n* = 2325).

	N	AstraZeneca	Janssen	Moderna	Pfizer	%
**Fever**	1265	256 (20.23)	3 (0.23)	78 (6.16)	928 (73.35)	36.3
**Pain at the injection site**	1772	257 (14.5)	3 (0.16)	79 (4.45)	1429 (80.64)	50.8
**Heat and swelling at the injection site**	592	106 (17.9)	1 (0.16)	32 (5.4)	374 (63.17)	17.0
**Fatigue**	1114	188 (16.87)	3 (0.26)	65 (5.83)	850 (76.3)	32.0
**muscle pain**	980	178 (18.16)	1 (0.1)	57 (5.81)	668 (68.16)	28.1
**Headache**	924	196 (21.21)	3 (0.32)	52 (5.62)	664 (71.86)	26.5
**Diarrhea**	67	23 (34.32)	0	2 (2.98)	38 (56.71)	3.0
**Rash**	28	1 (3.57)	0	3 (10.71)	24 (85.71)	0.9
**Stroke (heart, lung, brain)**	6	2 (33.33)	0	1 (16.67)	3 (50)	0.2
**No symptoms**	1054	217 (20.59)	4 (0.37)	11 (1.04)	815 (77.32)	30.2

## Supplementary Materials

The following are available online at https://www.mdpi.com/article/10.3390/ijerph192113889/s1. Table S1: The logistic univariate regression of the participants’ response with the acceptance of mix and match of different generic type of COVID-19 vaccine (*n* = 3486).
