# Supplementary material for: Perception and Acceptance of Using Different Generic Types of COVID-19 Vaccine, the “Mix-and-Match” Strategy, in Saudi Arabia: Cross-Sectional Web-Based Survey
Source: Int J Environ Res Public Health. 2022 Oct 26;19(21):13889. doi: 10.3390/ijerph192113889 (PMC9657038; doi:10.3390/ijerph192113889)
Supplement: Supplementary file 1 [file ijerph-19-13889-s001.zip › ijerph-1924390-supplementary.pdf]

Supplementary Table S1. The logistic univariate regression of the participants' response with the acceptance of mix and match of different generic type of COVID-19 vaccine (n=3486).

Participants' Response	**Taking the same generic types of COVID-19 vaccine**	
	OR	P-value
Receiving different types of corona vaccine causes severe side effects	-0.001 (-0.02-0.02)	0.89
Receiving different types of corona vaccine are not effective	-0.003 (-.03-0.02)	0.78
I will take two different types of vaccine if they are taken by many people	0.04 (0.02-0.07)	0.00*
I will take two different types of vaccine if advised by the doctor	0.05 (0.03-0.07)	0.00*
I will take two different types of vaccine if there is no alternative	0.022 (0.01-0.04)	0.01*
I will only take two different types of vaccines if vaccination is mandatory	-0.004 (-0.02-0.1)	0.62
I don't mind taking two different types of Coronaviruses (Covid-19) vaccines only if I have been given enough information about their safety	0.03 (0.01-0.05)	0.00*
Receiving two different types of vaccine reduces infection with (Covid-19) or its complications	0.03 (0.01-0.05)	0.00*
If I take two doses of the same type, I will take another type if a third dose is available	-0.007 (-0.02-0.01)	0.48
Would I advise others to receive the (Covid-19) vaccine of two different types?	0.04 (0.02-0.06)	0.00*
**OR: Odds Ratio, * significant difference**		
